# Spontaneous rupture of ovarian cystadenocarcinoma: pre- and post-rupture computed tomography evaluation*

**DOI:** 10.1590/0100-3984.2013.1844

**Published:** 2015

**Authors:** Priscila Silveira Salvadori, Lucas Novais Bomfim, Augusto Castelli von Atzingen, Giuseppe D’Ippolito

**Affiliations:** 1 Master, MD, Radiologist, Department of Imaging Diagnosis – Escola Paulista de Medicina da Universidade Federal de São Paulo (EPM-Unifesp), São Paulo, SP, Brazil.; 2 MD, Radiology Residency Preceptor at Hospital da Agro-Indústria do Açúcar e do Álcool de Alagoas, Professor of Imaging Sciences, Universidade Tiradentes (Unit), Maceió, AL, Brazil.; 3 PhD, MD, Radiologist, Department of Imaging Diagnosis – Escola Paulista de Medicina da Universidade Federal de São Paulo (EPM-Unifesp), São Paulo, SP, Associate Professor at Universidade Federal de Alfenas (Unifal), Alfenas, MG, Post-graduation Professor at Universidade do Vale do Sapucai' (Univa's), Pouso Alegre, MG, Brazil.; 4 Private Docent, Associate Professor, Department of Imaging Diagnosis – Escola Paulista de Medicina da Universidade Federal de São Paulo (EPM-Unifesp), São Paulo, SP, Brazil.

**Keywords:** Ovarian neoplasms, Serous cystadenocarcinoma, Spontaneous rupture

## Abstract

Epithelial ovarian tumors are the most common malignant ovarian neoplasms and, in most cases, eventual rupture of such tumors is associated with a surgical procedure. The authors report the case of a 54-year-old woman who presented with spontaneous rupture of ovarian cystadenocarcinoma documented by computed tomography, both before and after the event. In such cases, a post-rupture staging tends to be less favorable, compromising the prognosis.

## Abstract (pt)

Os tumores epiteliais correspondem à maioria das neoplasias ovarianas, sendo que eventuais rupturas estão mais relacionadas ao ato cirúrgico. Relatamos um caso de cistoadenocarcinoma ovariano em uma paciente de 54 anos com documentação tomográfica pré- e pós-ruptura espontânea. O estadiamento tumoral pós-ruptura tende a ser menos favorável, comprometendo o prognóstico desses pacientes.

## INTRODUCTION

Epithelial ovarian tumors correspond to the most lethal malignant neoplasia of the female genital tract. Mucinous and serous tumors are the two most common types of epithelial neoplasia^(1)^. Most commonly, rupture of ovarian cystadenocarcinoma is associated with surgical manipulation^(2)^, significantly changing the staging and prognosis of the patients.

Few reports are found in the literature about spontaneous rupture of ovarian cystadenocarcinoma and these reports are mainly regarding pregnant women^(3-5)^ and patients using anticoagulant drugs^(6)^. The authors report the first case of spontaneous rupture of ovarian serous cystadenocarcinoma documented by computed tomography both before and after the event.

## CASE REPORT

**Figure 1:**
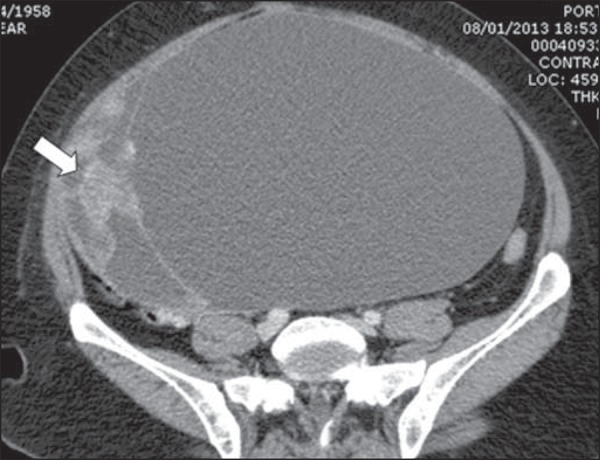
Voluminous complex cystic mass with solid component (arrow). Absence of ascites at that moment.

A 54-year-old woman attended the emergency department presenting with sudden-onset dyspnea associated with pain and increased abdominal volume. Chest computed tomography (CT) demonstrated the presence of pulmonary thromboembolism (PTE). Abdominal CT showed a large pelvic solid-cystic mass with gross septations and solid component, compatible with epithelial ovarian tumor (Figure 1).

**Figure 2:**
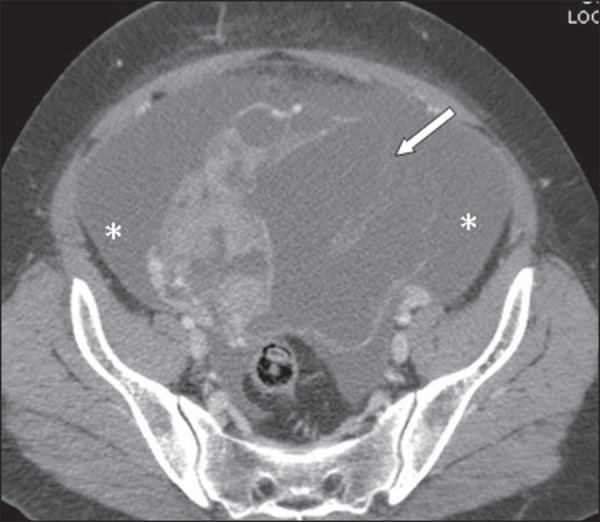
Reduction in dimensions of the cystic mass, parietal discontinuity (arrow) and voluminous ascites that was not identified at the previous study (asterisks) performed eight days ago.

The patient was admitted for treatment of the PTE, using heparin and, at the ninth day after the admission, she presented a sudden worsening in her condition with abdominal pain and a 3 g/dL drop in hemoglobin levels. A new abdominal CT revealed a voluminous ascites and reduction in the dimensions of the adnexal solid-cystic mass with parietal discontinuity, compatible with spontaneous rupture (Figure 2).

**Figure 3:**
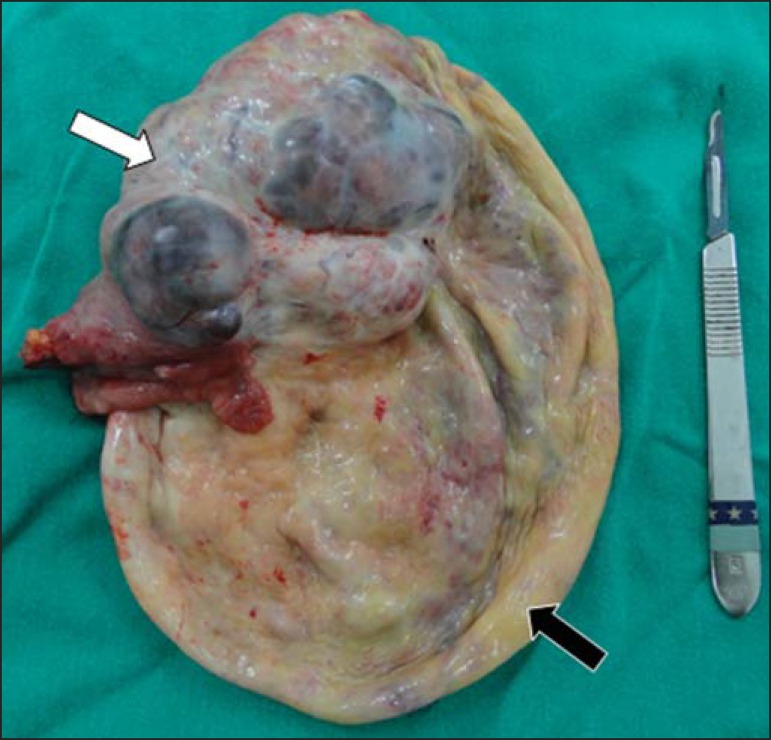
Surgical specimen with solid component (white arrow) and cystic component after the rupture (black arrow).

The patient was submitted to exploratory laparotomy that revealed a great amount of hematic ascites and an adnexal tumor with solid component and ruptured cystic area (Figure 3). Anatomopathological analysis characterized high-grade serous carcinoma.

## DISCUSSION

The most recent Brazilian population data analysis indicates a risk for ovarian cancer corresponding to 6 cases/100,000 women. The main risk factors for development of ovarian cancer include family or personal history of breast or ovarian cancer; post-menopausal hormone replacement therapy, smoking and obesity^(7)^. Epithelial ovarian tumors correspond to 60% of all ovarian neoplasms and 85% of the malignant ovarian neoplasias^(1)^.

Some of these tumors may present complications such as rupture, torsion, hemorrhage^(3)^ or metastasis^(8)^. Such tumors rupture is frequently reported as occurring intraoperatively in cases where a large lesion is attached to adjacent organs^(2)^. Spontaneous rupture rarely occurs, generally as a result either from internal tumor bleeding or from increased intralesional pressure in association with some risk factor^(3,4,6)^, such as anticoagulant therapies. A previous study reports spontaneous rupture of an ovarian cystadenocarcinoma in a patient with congestive heart failure and undergoing treatment with heparin who progressed with hemoperitoneum, characterized by the presence of hyperdense (65-70 Hounsfield units) fluid in the peritoneal cavity, but without clearly demonstrate the tumor lesion, probably because the scan was performed without using an intravenous contrast agent^(6)^. In the other cases described in the literature, imaging studies were not performed.

In the present case, the tumor lesion was clearly identified at the initial examination, presenting a sharp change in shape and reduction of dimensions at the subsequent scan, associated with the presence of ascites, which has allowed for establishing the diagnosis of tumor rupture, corroborated by the drop of hemoglobin levels resulting from the use of heparin due to the previous history of PTE. Heparin inhibits the Xa factor and thrombin, blocking the coagulation cascade at these levels with therapeutical purposes; hemorrhage is the main complication^(9)^. The authors believe that the heparin anticoagulation must have been responsible for a bleeding within the cyst, leading to increased intralesional pressure and subsequent rupture with hemoperitoneum.

Thus, the authors call the attention to the fact that the use of anticoagulant drugs in patients with voluminous cystic lesions must be done cautiously, taking the risk/benefit balance into consideration, with careful follow-up (by ultrasonography, CT or magnetic resonance imaging) along the treatment for evaluation of possible severe complications, like in the present case. Additionally, the extravasation of the cystic content may result in intraperitoneal malignant cells dissemination, modifying the staging, the approach to be adopted and the prognosis of the patient^(1)^.
